# Supplementary material for: Jag1/2 maintain esophageal homeostasis and suppress foregut tumorigenesis by restricting the basal progenitor cell pool
Source: Nat Commun. 2024 May 15;15:4124. doi: 10.1038/s41467-024-48347-5 (PMC11096375; doi:10.1038/s41467-024-48347-5)
Supplement: Supplementary file 1 — Supplementary Information [file 41467_2024_48347_MOESM1_ESM.pdf]

Supplementary Information

Jag1/2 maintain esophageal homeostasis and suppress foregut tumorigenesis by restricting the basal progenitor cell pool

Haidi Huang[1,12], Yu Jiang[1,12], Jiangying Liu[1], Dan Luo[1], Jianghong Yuan[1], Rongzi Mu[1], Xiang Yu[1], Donglei Sun[1], Jihong Lin[2], Qiyue Chen[3,4], Xinjing Li[1], Ming Jiang[5], Jianming Xu[6], Bo Chu[7], Chengqian Yin[8], Lei Zhang[8,9], Youqiong Ye[10], Bo Cao[1], Qiong Wang[11,*], Yongchun Zhang[1,*]

The PDF file includes:

Supplementary Figures 1 to 8

Supplementary Tables 1 to 3

Fig. S1

a

Mouse esophagus

Gene Symbol	WT1	WT2	WT3	WT4	WT5
Jag1	18.78	26.98	27.83	27.4	26.38
Jag2	20.4	15.32	15.23	20.04	17.36
Dll1	7.46	8.08	7.39	9.57	8.32
Dll3	0	0.02	0	0.02	0
Dll4	0	0	0	0	0

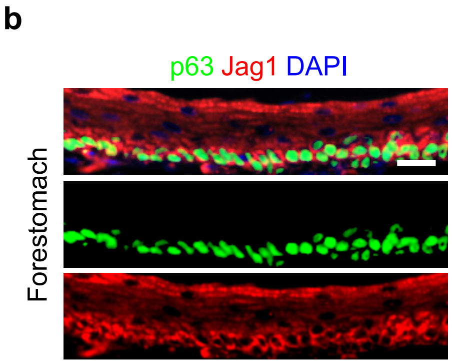

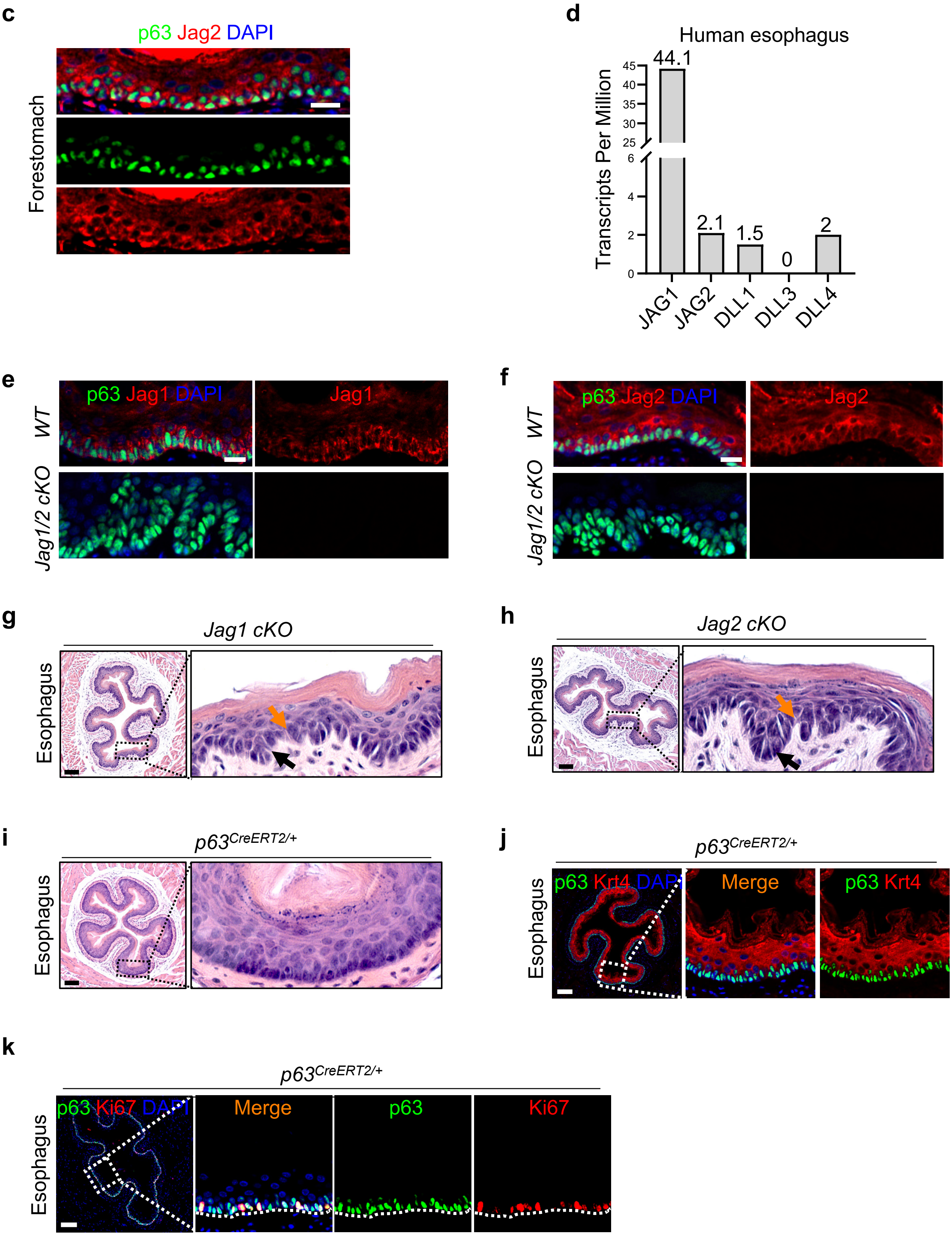

Supplementary Fig. 1. Jag1/2 deletion dysregulates homeostasis of the squamous epithelium in the esophagus. a Transcript levels in FPKM values of *Jag1/2* and *Dll1/3/4* in the esophageal epithelium of five wild type (*WT*) mice. FPKM, Fragments Per Kilobase of transcript per Million mapped reads (n = 5). **b, c** Immunofluorescence staining of p63, Jag1 (**b**) and Jag2 (**c**) in the forestomach epithelium of *WT* mice. Note the high expression of Jag1/2 in the basal cells, and the staining observed in the superficial layer is nonspecific marginal staining. Representative images are shown (n = 3 in b, n = 3 in c). Scale bars: 20 µm. **d** Transcript levels of *Jag1/2* and *Dll1/3/4* in the normal human esophagus. **e, f** Immunostaining showed efficient deletion of Jag1 (**e**) Jag2 (**f**) in the esophageal epithelium of *Jag1/2 cKO* mice. Representative images are shown (n = 4 in e per genotype, n = 4 in f per genotype). Scale bars: 20 µm. **g, h** Representative H&E stained esophageal sections (n = 3 in g, n = 3 in h). Note the increased basal cell number (darker blue nuclei, black arrows) and intercellular space (orange arrows) in the esophageal epithelium of *Jag1 cKO* and *Jag2 cKO* mice (orange arrows). Scale bars: 100 µm. *WT*, *wild type*; *Jag1 cKO*, *p63*$^{CreERT2/+}$*;Jag1*$^{loxp/loxp}$. *Jag2 cKO*, *p63*$^{CreERT2/+}$*;Jag2*$^{loxp/loxp}$. **i-k** H&E (**i**) and immunofluorescence staining (**j, k**) showed no phenotypic changes in the esophageal epithelium of *p63*$^{CreERT2/+}$mice. Representative images are shown (n = 6). Scale bars: 100 µm.

Fig. S2

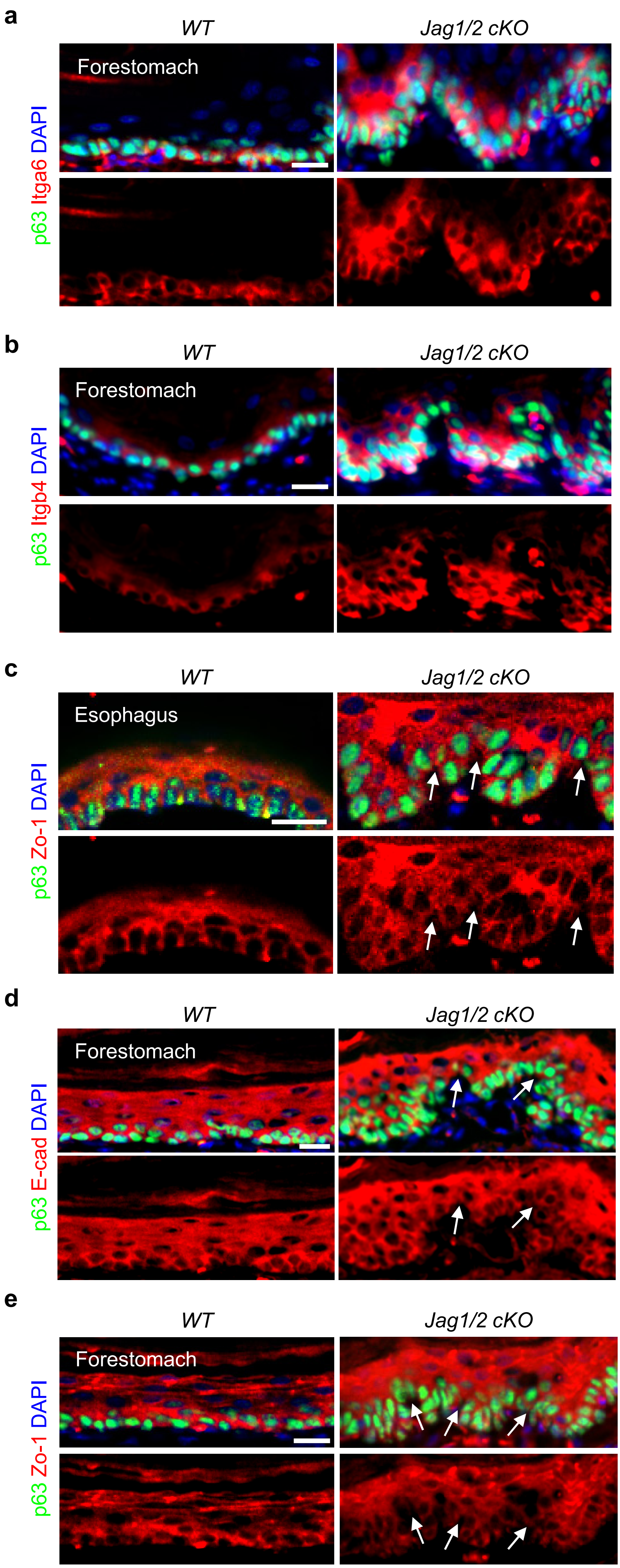
a
WT
Jag1/2 cKO
Forestomach
p63 Itga6 DAPI
b
WT
Jag1/2 cKO
Forestomach
p63 Itgb4 DAPI
c
WT
Jag1/2 cKO
Esophagus
p63 Zo-1 DAPI
d
WT
Jag1/2 cKO
Forestomach
p63 E-cad DAPI
e
WT
Jag1/2 cKO
Forestomach
p63 Zo-1 DAPI

Supplementary Fig. 2. Jag1/2 deletion results in increased expression of basal cell adhesion molecules in the forestomach epithelium and dilated intercellular space in the esophageal and forestomach epithelium. a, b Immunofluorescence staining of p63, Itga6, and Itgb4 in the forestomach epithelium. Note the increased expression of Itga6 and Itgb4 in the *Jag1/2 cKO* mutants. **c-e** Immunofluorescence staining of p63, Zo-1, and E-cadherin in the esophageal or forestomach epithelium. Note the dilated intercellular space in both the esophageal and forestomach epithelium of *Jag1/2 cKO* mutants. Representative images in a-e are shown (n = 3). *WT*, *wild type*; *Jag1/2 cKO*, *p63* *p63*$^{CreERT2/+}$*;Jag1/2*$^{loxp/loxp}$. Scale bars: 20 µm.

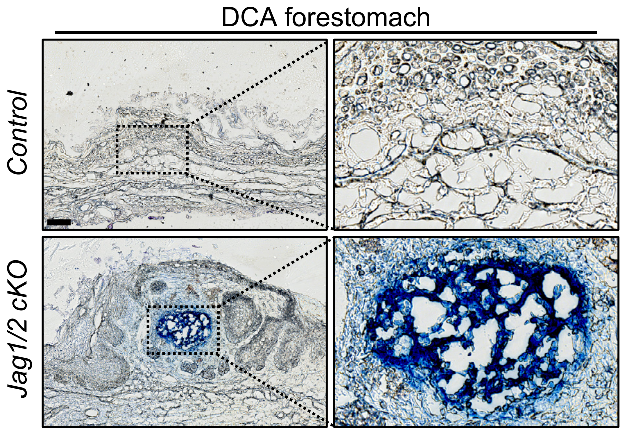

Supplementary Fig. 3. Evans blue staining of the forestomach of deoxycholic acid (DCA)-treated mice. Note the increased Evans blue staining within the forestomach mucosa of the *p63*$^{CreERT2/+}$*;Jag1/2*$^{loxp/loxp}$ (*Jag1/2 cKO*) mutants. Representative images are shown (n = 3). Scale bar: 100 µm.

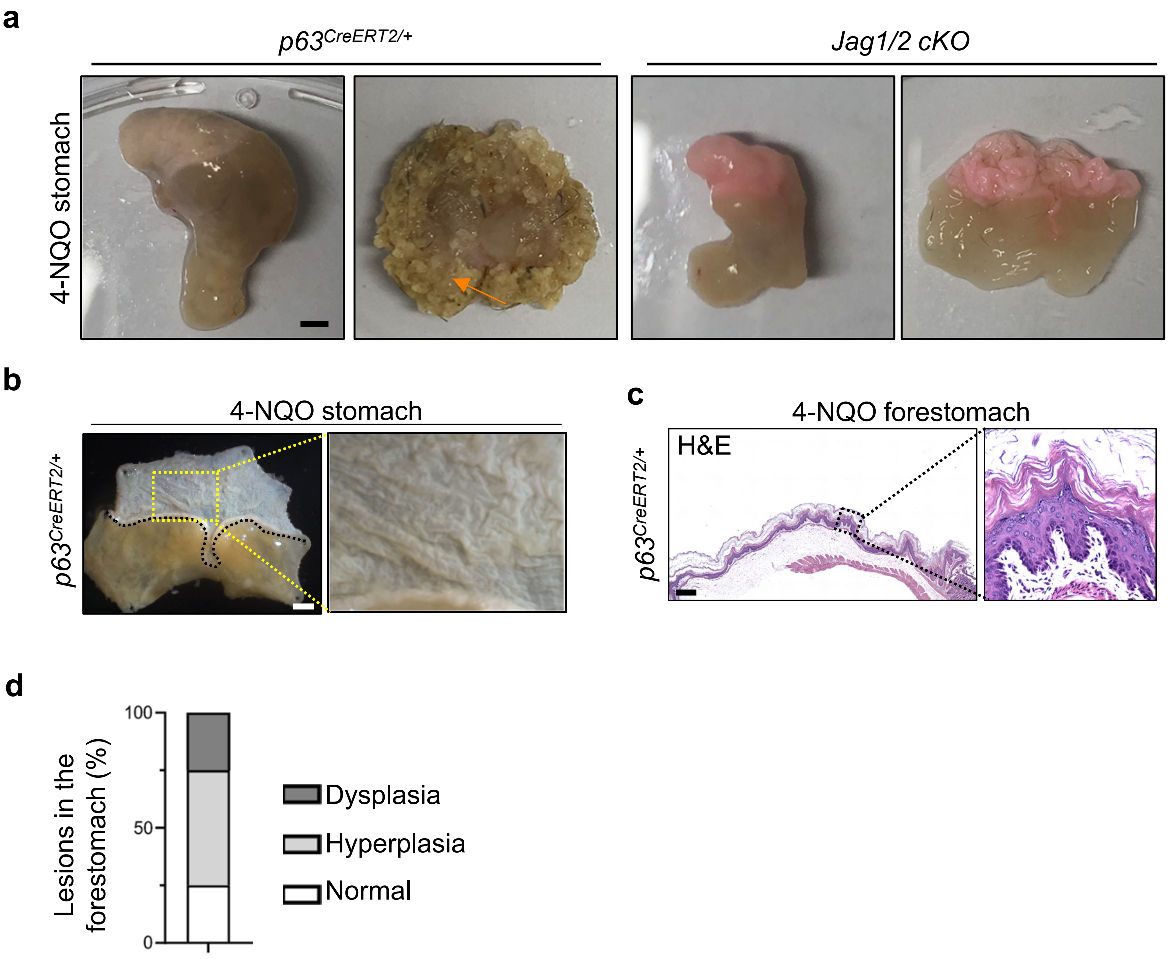

Supplementary Fig. 4. Stomach of the 4-NQO-treated mice. a The control *p63*$^{CreERT2/+}$ mice showed food (arrows) within the stomach, while no food was observed in the *p63*$^{CreERT2/+}$*;Jag1/2*$^{loxp/loxp}$ (*Jag1/2 cKO*) mutants when 4-NQO-induced SCC developed in the forestomach. Representative images are shown (n = 4). **b-d** Representative gross morphology (**b**), H&E-stained sections (**c**), and the percentage of lesions (**d**) in *p63*$^{CreERT2/+}$mice fed with 4-NQO water. Representative images in b, c are shown (n = 4). Scale bars: a, 20 mm; b, 2 mm; c, 200 µm.

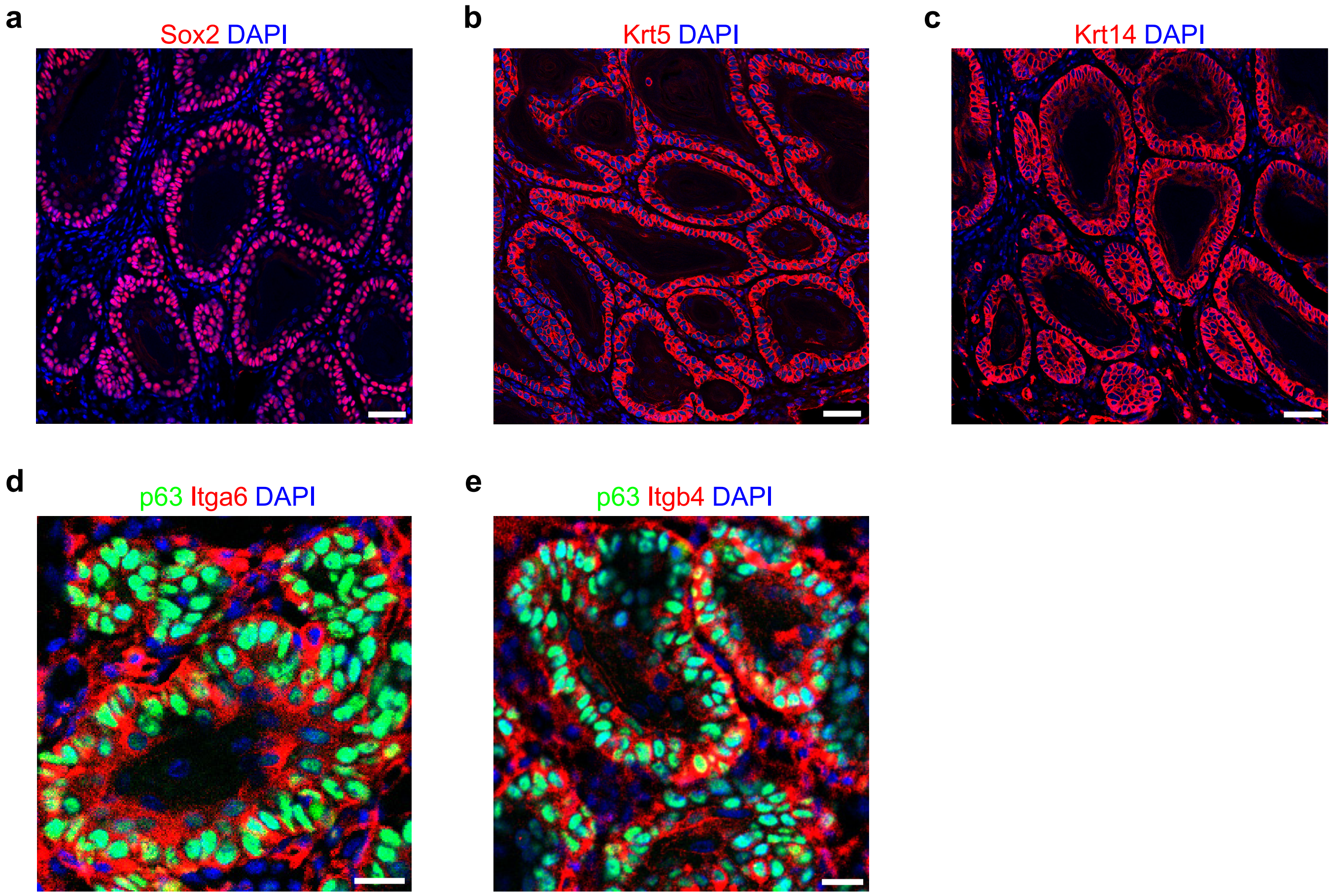

Supplementary Fig. 5. Forestomach squamous cell carcinoma (SCC) induced by 4-NQO in *Jag1/2 cKO* mice expresses high levels of SCC diagnostic markers SOX2, Krt5, Krt14, p63, Itga6, and Itgb4. Immunofluorescence staining of Sox2 (**a**), Krt5 (**b**), Krt14 (**c**), p63 and Itga6 (**d**), p63 and Itgb4 (**e**) in the 4-NQO-induced forestomach SCC of *Jag1/2 cKO* mice. Representative images in a-e are shown (n = 5). Scale bars in a-c, 50 µm; d-e, 20 µm.

Fig. S6

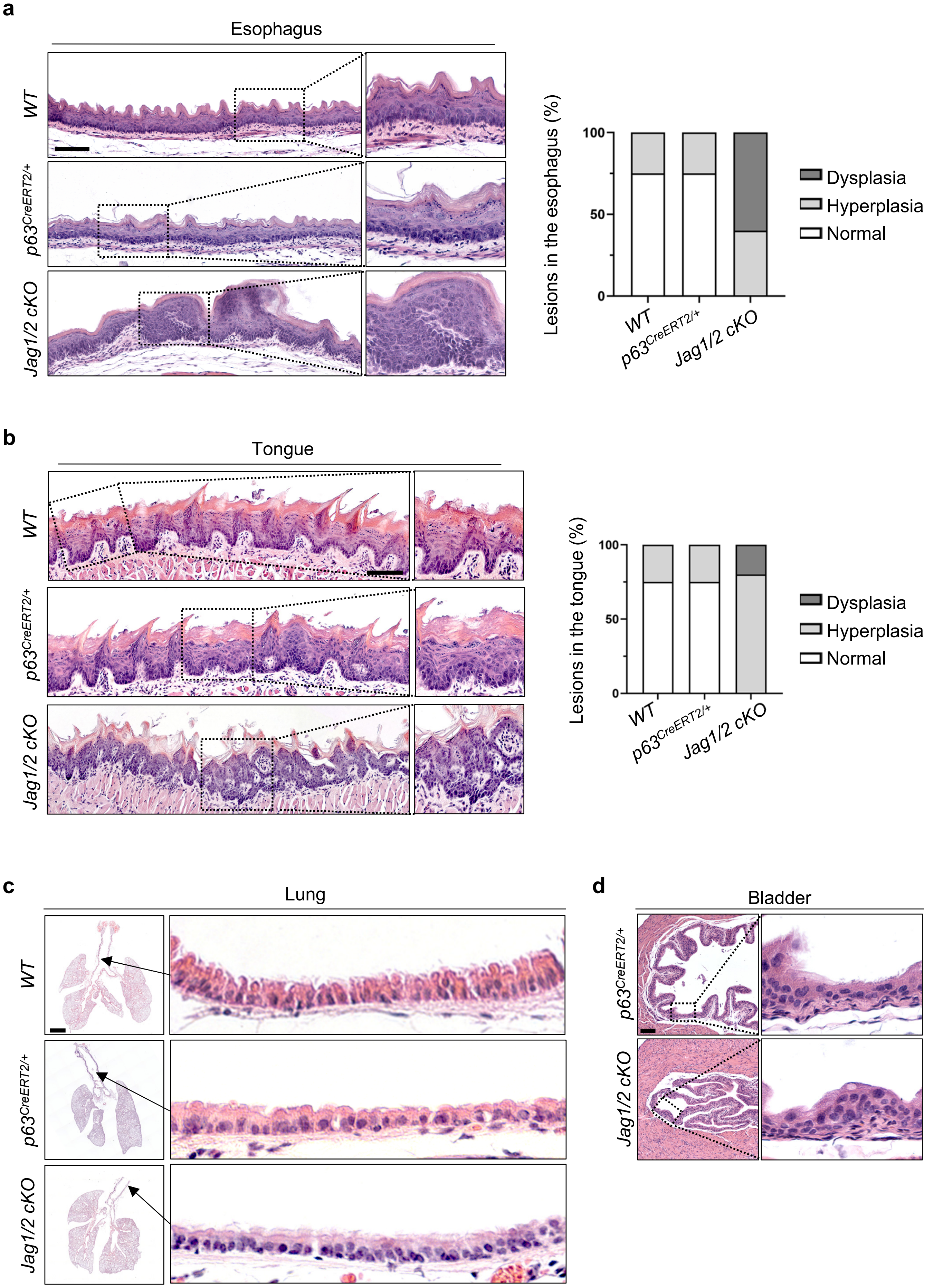
a
Esophagus
WT
p63CreERT2/+
Jag1/2 cKO
Lesions in the esophagus (%)
100
50
0
WT
p63CreERT2/+
Jag1/2 cKO
Dysplasia
Hyperplasia
Normal
b
Tongue
WT
p63CreERT2/+
Jag1/2 cKO
Lesions in the tongue (%)
100
50
0
WT
p63CreERT2/+
Jag1/2 cKO
Dysplasia
Hyperplasia
Normal
c
Lung
WT
p63CreERT2/+
Jag1/2 cKO
d
Bladder
p63CreERT2/+
Jag1/2 cKO

Supplementary Fig. 6. Histological analysis of the esophagus, tongue, lung, and bladder. a, b *p63*$^{CreERT2/+}$;*Jag1/2*$^{loxp/loxp}$ (*Jag1/2 cKO*) showed more severe dysplasia in the esophagus and tongue compared to the control *WT* or *p63*$^{CreERT2/+}$ mice (n = 4 for *WT*; n = 4 for *p63*$^{CreERT2/+}$; n = 5 for *Jag1/2 cKO*). Scale bars: 100 µm. **c, d** No obvious phenotypic changes were observed in the lung and bladder *Jag1/2 cKO* and *WT* or *p63*$^{CreERT2/+}$ mice. Representative images are shown (n = 4 in c per genotype, n = 4 in d per genotype). Scale bars: c, 2 mm; d, 100 µm.

Fig. S7

a

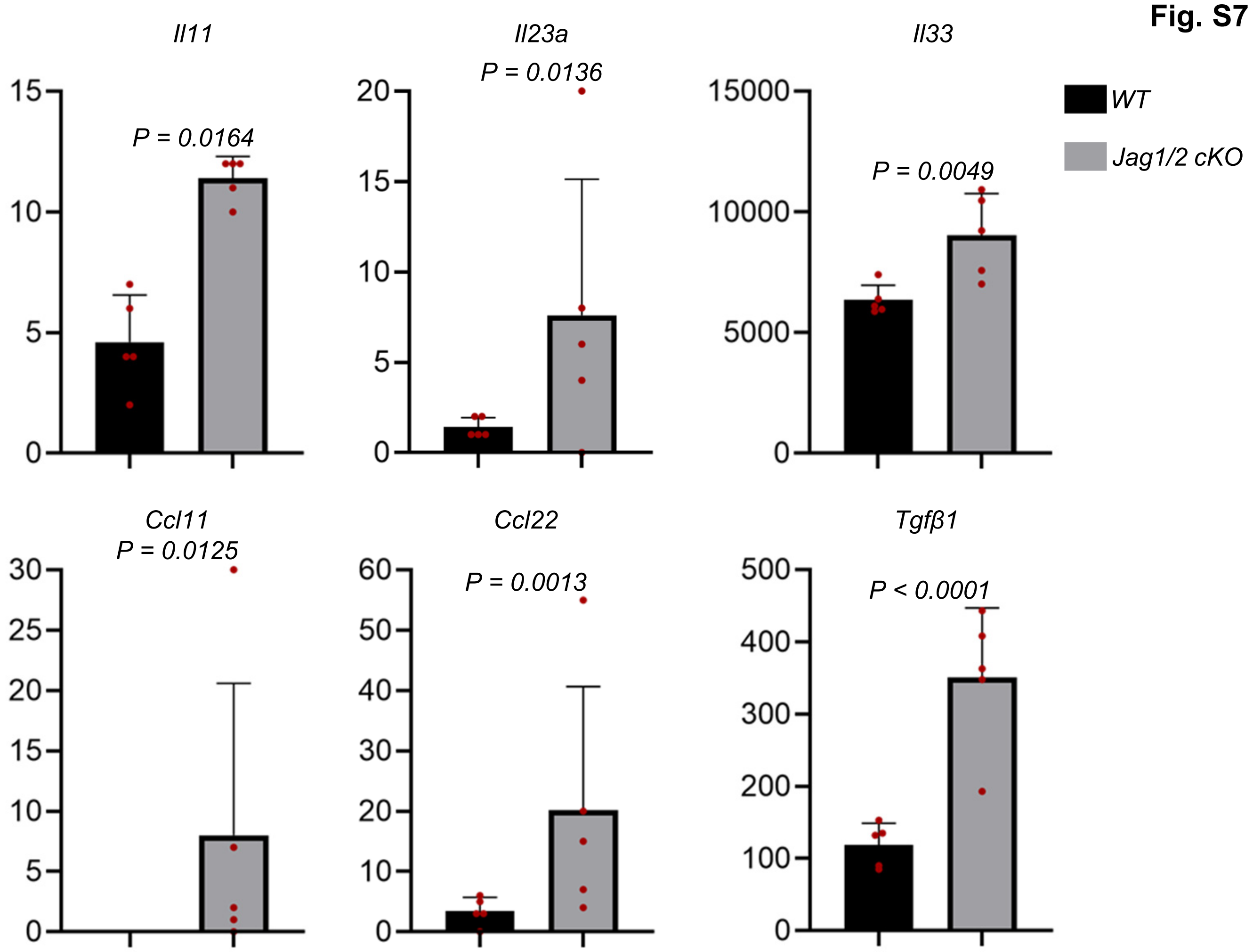

b

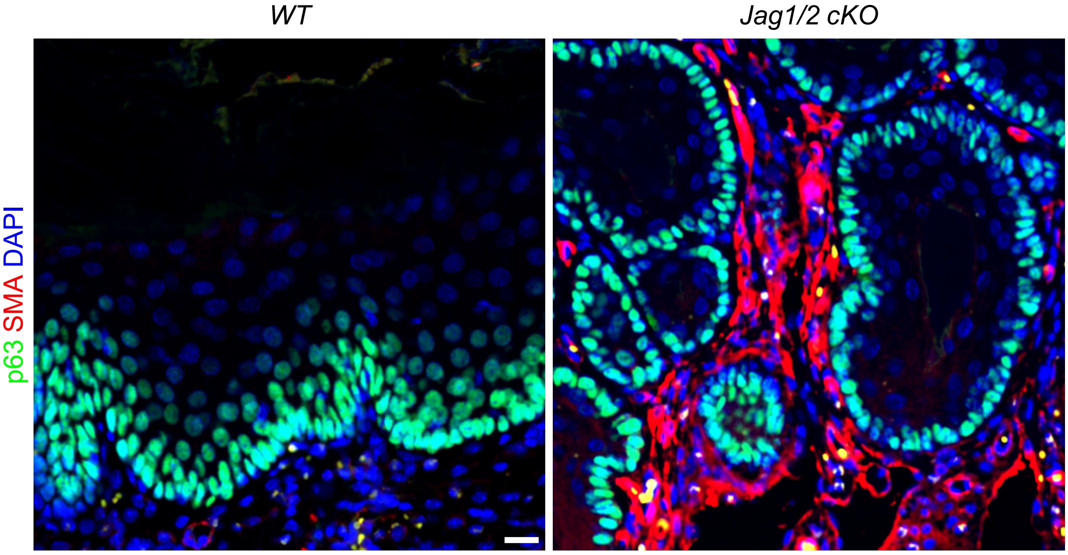

Supplementary Fig. 7. Pro-inflammatory cytokines are increased in *Jag1/2 cKO* esophageal epithelium, and tumor cells in the SCC of *Jag1/2 cKO* mice are surrounded by stromal fibroblasts. a Transcript levels of *Il11, Il23a, Il33, Ccl11, Ccl22,* and *Tgfβ1* in the *Jag1/2 cKO* mouse esophageal epithelium during homeostasis. Transcript levels were determined by RNA sequencing and presented in read counts. Data represent mean ± SD (n = 5 per genotype). P values were determined by the two-tailed Wald test. **b** Immunofluorescence staining of p63 and smooth muscle actin (SMA) in the 4-NQO-induced forestomach SCC. Representative images are shown (n = 4 per genotype). Scale bar: 20 µm.

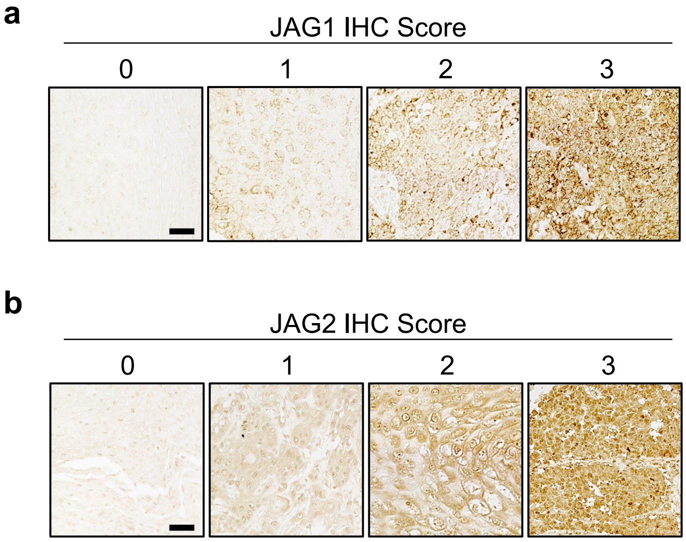

Supplementary Fig. 8. Immunohistochemistry (IHC) scores of JAG1 and JAG2 in the human esophageal samples. Representative IHC images of JAG1 **(a)** and JAG2 **(b)** of each score in human esophageal squamous cell carcinoma samples or the normal esophageal epithelium. Representative images are shown (n = 3). Scale bars: 50 µm.

Supplementary Table 1. Primary antibody list

Primary antibody name	Source	Catalog number and RRID	Dilution
Rabbit monoclonal anti-p63	Cell Signaling Technology	Cat#13109S, RRID: AB_2637091	IF: 1:200
Rat monoclonal anti-Ki67	Thermo Fisher Scientific	Cat#14-5698-82, RRID: AB_10854564	IF: 1:200
Mouse monoclonal anti- KRT4	Santa Cruz Biotechnology	Cat#sc-52321, RRID: AB_2249751	IF: 1:200; WB: 1:500
Mouse monoclonal anti-Jagged1	Santa Cruz Biotechnology	Cat#sc-390177, RRID: AB_2892141	IF and IHC: 1:200
Armenian Hamster monoclonal anti-mouse Jagged2	BioLegend	Cat#131001, RRID: AB_1227682	IF and IHC: 1:200
Mouse monoclonal anti-ITGA6	Santa Cruz Biotechnology	Cat#sc-374057, RRID: AB_10917002	IF: 1:200
Mouse monoclonal anti-ITGB1	Santa Cruz Biotechnology	Cat#sc-9970, RRID: AB_627004	IF: 1:200
Mouse monoclonal anti-PKC zeta	Santa Cruz Biotechnology	Cat#sc-17781, RRID: AB_628148	IF: 1:200
Rat monoclonal anti-E-cadherin	Sigma-Aldrich	Cat#U3254, RRID: AB_477600	IF: 1:200
Mouse monoclonal PE anti-human CD104 (ITGB4)	BioLegend	Cat#327807, RRID: AB_2129147	IF: 1:200
Chicken polyclonal anti-KRT5	BioLegend	Cat#905901, RRID: AB_2565054	IF: 1:200
Rat monoclonal purified anti-mouse F4/80	BioLegend	Cat#123102, RRID: AB_893506	IF: 1:200
Mouse monoclonal purified anti-human CD68	BioLegend	Cat#375602, RRID: AB_2876705	IF: 1:200
Rat monoclonal purified anti-mouse Ly6G	BioLegend	Cat#127602, RRID: AB_1089180	IF: 1:200
Rat monoclonal purified anti-mouse CD4	BioLegend	Cat#100402, RRID: AB_312687	IF: 1:200
Rat monoclonal purified anti-mouse CD8a	BioLegend	Cat#100702, RRID: AB_312741	IF: 1:200
Rat monoclonal anti-ZO-1	Santa Cruz Biotechnology	Cat#sc-33725, RRID: AB_628459	IF: 1:200
Mouse monoclonal anti-SMA	Santa Cruz Biotechnology	Cat#sc-53142, RRID: AB_2273670	IF: 1:200
Mouse monoclonal anti-KRT14	Thermo Fisher Scientific	Cat#MA5-11599, RRID: AB_10982092	IF: 1:200
Rat monoclonal anti-SOX2	Thermo Fisher Scientific	Cat#14-9811-82; RRID: AB_11219471	IF: 1:200
Rabbit monoclonal anti-Cleaved Notch1 (NICD1)	Cell Signaling Technology	Cat#4147; RRID: AB_2153348	WB: 1:1000
Mouse monoclonal anti-Notch1	Santa Cruz Biotechnology	Cat#sc-376403, RRID: AB_11149738	IF: 1:200
Mouse monoclonal anti-GAPDH	Proteintech	Cat#60004-1, RRID: AB_2107436	WB: 1:3000

Supplementary Table 2. Secondary antibody list

Secondary antibody name	Source	Catalog number	Dilution
Alexa Fluor 488 AffiniPure™ Donkey Anti-Rat IgG	Jackson ImmunoResearch Laboratories	Cat#712-545-153	IF: 1:500
Alexa Fluor 488 AffiniPure™ Donkey Anti-Rabbit IgG	Jackson ImmunoResearch Laboratories	Cat#711-545-152	IF: 1:500
Alexa Fluor 488 AffiniPure™ Donkey Anti-Mouse IgG	Jackson ImmunoResearch Laboratories	Cat#715-545-151	IF: 1:500
Alexa Fluor 488 AffiniPure™ Goat Anti-Armenian Hamster IgG	Jackson ImmunoResearch Laboratories	Cat#127-545-099	IF: 1:500
Cy™3 AffiniPure™ Donkey Anti-Mouse IgG	Jackson ImmunoResearch Laboratories	Cat#715-165-151	IF: 1:500
Cy™3 AffiniPure™ Donkey Anti-Rat IgG	Jackson ImmunoResearch Laboratories	Cat#712-165-150	IF: 1:500
Cy™3 AffiniPure™ Donkey Anti-Rabbit IgG	Jackson ImmunoResearch Laboratories	Cat#711-165-152	IF: 1:500
Cy™5 AffiniPure™ Donkey Anti-Mouse IgG	Jackson ImmunoResearch Laboratories	Cat#715-175-151	IF: 1:500
Cy™5 AffiniPure™ Donkey Anti-Rat IgG	Jackson ImmunoResearch Laboratories	Cat#712-175-153	IF: 1:500
Cy™5 AffiniPure™ Donkey Anti-Rabbit IgG	Jackson ImmunoResearch Laboratories	Cat#711-175-152	IF: 1:500
Cy™5 AffiniPure™ Donkey Anti-Chicken IgG	Jackson ImmunoResearch Laboratories	Cat#703-175-155	IF: 1:500
Mouse anti-Armenian hamster IgG-HRP	Santa Cruz Biotechnology	Cat#sc-2789	IHC: 1:500
HRP-conjugated Goat anti-Rabbit IgG Secondary Antibody	Thermo Fisher Scientific	Cat#31460	WB: 1:3000
HRP-conjugated Goat anti-Mouse IgG Secondary Antibody	Thermo Fisher Scientific	Cat#31430	IHC:1:500; WB: 1:3000

Supplementary Table 3. qPCR Primer list

Gene	Forward primer	Reverse primer
Mouse Krt4	AGAACCTGGACACTTTGAGC	ACCACGAAGTCATTCTCTGC
Mouse p63	GAGACGTACGAGATGTTGCTG	GAAGACTGAGACTGCATCGAG
