# Supplementary material for: Jag1/2 maintain esophageal homeostasis and suppress foregut tumorigenesis by restricting the basal progenitor cell pool
Source: Nat Commun. 2024 May 15;15:4124. doi: 10.1038/s41467-024-48347-5 (PMC11096375; doi:10.1038/s41467-024-48347-5)
Supplement: Supplementary file 2 — Reporting Summary [file 41467_2024_48347_MOESM2_ESM.pdf]

nature portfolio

Corresponding author(s): Yongchun Zhang

Last updated by author(s): Mar 14, 2024

Reporting Summary

Nature Portfolio wishes to improve the reproducibility of the work that we publish. This form provides structure for consistency and transparency in reporting. For further information on Nature Portfolio policies, see our Editorial Policies and the Editorial Policy Checklist.

Statistics

For all statistical analyses, confirm that the following items are present in the figure legend, table legend, main text, or Methods section.

n/a	Confirmed	
☐	☒	The exact sample size (n) for each experimental group/condition, given as a discrete number and unit of measurement
☐	☒	A statement on whether measurements were taken from distinct samples or whether the same sample was measured repeatedly
☐	☒	The statistical test(s) used AND whether they are one- or two-sided<br>*Only common tests should be described solely by name; describe more complex techniques in the Methods section.*
☒	☐	A description of all covariates tested
☐	☒	A description of any assumptions or corrections, such as tests of normality and adjustment for multiple comparisons
☐	☒	A full description of the statistical parameters including central tendency (e.g. means) or other basic estimates (e.g. regression coefficient) AND variation (e.g. standard deviation) or associated estimates of uncertainty (e.g. confidence intervals)
☐	☒	For null hypothesis testing, the test statistic (e.g. F, t, r) with confidence intervals, effect sizes, degrees of freedom and P value noted<br>*Give P values as exact values whenever suitable.*
☒	☐	For Bayesian analysis, information on the choice of priors and Markov chain Monte Carlo settings
☒	☐	For hierarchical and complex designs, identification of the appropriate level for tests and full reporting of outcomes
☐	☒	Estimates of effect sizes (e.g. Cohen's d, Pearson's r), indicating how they were calculated

Our web collection on statistics for biologists contains articles on many of the points above.

Software and code

Policy information about availability of computer code

Data collection	Illumina NovaSeq 6000 was used for RNA sequencing data collection.<br>ZEISS Axio Imager 2 microscope was used for hematoxylin and eosin-stained tissue data collection.<br>Olympus MVX10 stereoscopic microscope was used for imaging the gross morphology of large tissues.<br>Nikon Ni-E A1 HD25 confocal microscope was used for imaging immunostaining tissues.<br>Thermo Fisher Scientific Talos L120C G2 transmission electron microscope was used for acquiring transmission electron microscopy images.<br>The real-time quantitative PCR data was collected by the qTOWER³ real-time PCR Thermal Cyclers (Analytik Jena).<br>Western blot data were collected by the ChemiDoc Imaging system (Bio-Rad).
Data analysis	GraphPad Prism 9 (v9.0.0.121) was used for statistical analyses.<br>ImageJ (National Institutes of Health) (v1.53c) was used for the quantitative analysis of the immunofluorescence-stained images.<br>Cutadapt (v1.9.1), HISAT2 (v2.0.1), and HTSeq (v0.6.1) were used for processing RNA-seq data and statistical analyses.

For manuscripts utilizing custom algorithms or software that are central to the research but not yet described in published literature, software must be made available to editors and reviewers. We strongly encourage code deposition in a community repository (e.g. GitHub). See the Nature Portfolio guidelines for submitting code & software for further information.

Data

Policy information about availability of data

All manuscripts must include a data availability statement. This statement should provide the following information, where applicable:

- Accession codes, unique identifiers, or web links for publicly available datasets
- A description of any restrictions on data availability
- For clinical datasets or third party data, please ensure that the statement adheres to our policy

The RNA-seq data generated in this study have been deposited in the Genome Sequence Archive in National Genomics Data Center, China National Center for Bioinformation / Beijing Institute of Genomics, Chinese Academy of Sciences database under accession code CRA011089 (https://ngdc.cncb.ac.cn/gsa). The UCSC/mm9 mouse reference genome used in this study is available in http://genome.ucsc.edu/cgi-bin/hgGateway?db=mm9. All data of this study are available in the published article and Supplementary Information. Source data are provided with this paper.

Research involving human participants, their data, or biological material

Policy information about studies with human participants or human data. See also policy information about sex, gender (identity/presentation), and sexual orientation and race, ethnicity and racism.

Reporting on sex and gender	Human esophageal squamous cell carcinoma (ESCC) samples and adjacent normal esophageal epithelial tissues were collected at Fujian Medical University Union Hospital from 50–78-year-old male and female ESCC patients. The study was approved by the ethics committee of Fujian Medical University Union Hospital.
Reporting on race, ethnicity, or other socially relevant groupings	N/A
Population characteristics	50–78-year-old ESCC patients in the Fujian Medical University Union Hospital.
Recruitment	All samples were obtained with informed consent from all subjects and in accordance with the ethical standards of the same institute.
Ethics oversight	The study was approved by the ethics committee of Fujian Medical University Union Hospital.

Note that full information on the approval of the study protocol must also be provided in the manuscript.

Field-specific reporting

Please select the one below that is the best fit for your research. If you are not sure, read the appropriate sections before making your selection.

☒ Life sciences ☐ Behavioural & social sciences ☐ Ecological, evolutionary & environmental sciences

For a reference copy of the document with all sections, see nature.com/documents/nr-reporting-summary-flat.pdf

Life sciences study design

All studies must disclose on these points even when the disclosure is negative.

Sample size	Sample sizes were chosen based on previously published works of a similar nature (Mol Cancer Res. 2006 Jun;4(6):401-10; Nat Genet. 2020 Jun;52(6):604-614. FEBS J. 2006 May;273(9):1930-47; Nat Commun. 2022 Oct 20;13(1):6206; Nat Commun. 2019 May 20;10(1):2225). Exact sample size, error bars, and statistical tests were utilized based on the experiments and are stated in the figure legends. All the experiments were performed at least three times. No statistical methods were used to pre-determine sample sizes.
Data exclusions	No data was excluded.
Replication	All experiments were replicated at least 3 times. The results were reliably reproduced.
Randomization	No specific methods were used to randomly allocate samples to groups. For mouse experiments, mice were allocated into different groups based on defined genotypes.
Blinding	The investigators were blind at the time of experiment execution, data acquisition, and image quantification. Blinding was not possible for mice, as mice of defined genotypes needed to be assigned to different groups.

Reporting for specific materials, systems and methods

We require information from authors about some types of materials, experimental systems and methods used in many studies. Here, indicate whether each material, system or method listed is relevant to your study. If you are not sure if a list item applies to your research, read the appropriate section before selecting a response.

Materials & experimental systems

n/a	Involved in the study
☐	☒ Antibodies
☒	☐ Eukaryotic cell lines
☒	☐ Palaeontology and archaeology
☐	☒ Animals and other organisms
☒	☐ Clinical data
☒	☐ Dual use research of concern
☒	☐ Plants

Methods

n/a	Involved in the study
☒	☐ ChIP-seq
☒	☐ Flow cytometry
☒	☐ MRI-based neuroimaging

Antibodies

Antibodies used

Name, Supplier, Catalog, Clone name, Lot number, with dilution
1. Rabbit monoclonal anti-human p63, Cell Signaling Technology, Cat:13109S, Clone name: D2K8X, Lot: 3, 1:200 dilution for immunofluorescence (IF) staining.
2. Rat monoclonal anti-Ki67, Thermo Fisher Scientific, Cat:14-5698-82, Clone name: SolA15, Lot: 2355034, 1:200 dilution for IF.
3. Mouse monoclonal anti-human KRT4, Santa Cruz Biotechnology, Cat: sc-52321, Clone name: 6B10, Lot: C0119, 1:200 dilution for IF, 1: 500 western blot (WB).
4. Mouse monoclonal anti-human Jagged1, Santa Cruz Biotechnology, Cat: sc-390177, Clone name: E-12, Lot: F0921, 1:200 dilution for IF and immunohistochemistry (IHC) staining.
5. Armenian Hamster monoclonal anti-mouse Jagged2, BioLegend, Cat: 131001, Clone name: HMJ2-1, Lot: B260931, 1:200 dilution for IF and IHC.
6. Mouse monoclonal anti-human ITGA6, Santa Cruz Biotechnology, Cat: sc-374057, Clone name: F-6, Lot: A0723, 1:200 dilution for IF.
7. Mouse monoclonal anti-human ITGB1, Santa Cruz Biotechnology, Cat: sc-9970, Clone name: 4B7R, Lot: F0623, 1:200 dilution for IF.
8. Mouse monoclonal anti-rat PKC zeta, Santa Cruz Biotechnology, Cat: sc-17781, Clone name: H-1, Lot: D2921, 1:200 dilution for IF.
9. Mouse monoclonal PE anti-human CD104 (ITGB4), BioLegend, Cat: 327807, Clone name: 58XB4, Lot: B248520, 1:200 dilution for IF.
10. Rat monoclonal anti-E-cadherin, Sigma-Aldrich, Cat: U3254, Clone name: DECMA-1, 1:200 dilution for IF.
11. Chicken polyclonal anti-KRT5, BioLegend, Cat: 905901, Clone name: Poly9059, Lot: B331577, 1:200 dilution for IF.
12. Rat monoclonal Purified anti-mouse F4/80, BioLegend, Cat: 123102, Clone name: BM8, Lot: B331496, 1:200 dilution for IF.
13. Mouse monoclonal Purified anti-human CD68, BioLegend, Cat: 375602, Clone name: BL13756, Lot: B343895, 1:200 dilution for IF.
14. Rat monoclonal Purified anti-mouse Ly-6G, BioLegend, Cat: 127602, Clone name: 1A8, Lot: B349412, 1:200 dilution for IF.
15. Rat monoclonal Purified anti-mouse CD4, BioLegend, Cat: 100402, Clone name: GK1.5, Lot: B287214, 1:200 dilution for IF.
16. Rat monoclonal Purified anti-mouse CD8a, BioLegend, Cat: 100702, Clone name: 53-6.7, Lot: B341791, 1:200 dilution for IF.
17. Rat monoclonal anti-ZO-1, Santa Cruz Biotechnology, Cat: sc-33725, Clone name: R40.76, Lot: L1621, 1:200 dilution for IF.
18. Mouse monoclonal anti-SMA, Santa Cruz Biotechnology, Cat: sc-53142, Clone name: B4, Lot: G2122, 1:200 dilution for IF.
19. Mouse monoclonal anti-KRT14, Thermo Fisher Scientific, Cat: MA5-11599, Clone name: LL002, 1:200 dilution for IF.
20. Mouse monoclonal anti-Notch1, Santa Cruz Biotechnology, Cat: sc-376403, Clone name: A-8, Lot: L1922, 1:200 dilution for IF.
21. Rat monoclonal anti-SOX2, Thermo Fisher Scientific, Cat:14-9811-82,Clone name:Btjce, 1:200 dilution for IF.
22. Mouse monoclonal anti-GAPDH, Proteintech, Cat: 60004-1-lg, Clone name: 1E6D9, Lot: 10028230, 1:3000 dilution for western blot (WB).
23. Rabbit monoclonal anti-Cleaved Notch1 (NICD1), Cell Signaling Technology, Cat: 4147, Clone name: D3B8, Lot: 11, 1:1000 dilution for WB.
24. Alexa Fluor 488 AffiniPure Donkey Anti-Rat IgG, Jackson ImmunoResearch Laboratories, Cat: 712-545-153, 1:500 dilution for IF.
25. Alexa Fluor 488 AffiniPure Donkey Anti-Rabbit IgG, Jackson ImmunoResearch Laboratories, Cat: 711-545-152, 1:500 dilution for IF.
26. Alexa Fluor 488 AffiniPure Donkey Anti-Mouse IgG, Jackson ImmunoResearch Laboratories, Cat: 715-545-151, 1:500 dilution for IF.
27. Alexa Fluor 488 AffiniPure Goat Anti-Armenian Hamster IgG, Jackson ImmunoResearch Laboratories, Cat: 127-545-099, 1:500 dilution for IF.
28. Cy3 AffiniPure Donkey Anti-Mouse IgG, Jackson ImmunoResearch Laboratories, Cat: 715-165-151, 1:500 dilution for IF.
29. Cy3 AffiniPure Donkey Anti-Rat IgG, Jackson ImmunoResearch Laboratories, Cat: 712-165-150, 1:500 dilution for IF.
30. Cy3 AffiniPure Donkey Anti-Rabbit IgG, Jackson ImmunoResearch Laboratories, Cat: 711-165-152, 1:500 dilution for IF.
31. Cy5 AffiniPure Donkey Anti-Mouse IgG, Jackson ImmunoResearch Laboratories, Cat: 715-175-151, 1:500 dilution for IF.
32. Cy5 AffiniPure Donkey Anti-Rat IgG, Jackson ImmunoResearch Laboratories, Cat: 712-175-153, 1:500 dilution for IF.
33. Cy5 AffiniPure Donkey Anti-Rabbit IgG, Jackson ImmunoResearch Laboratories, Cat: 711-175-152, 1:500 dilution for IF.
34. Cy5 AffiniPure Donkey Anti-Chicken IgG, Jackson ImmunoResearch Laboratories, Cat: 703-175-155, 1:500 dilution for IF.
35. Mouse anti-Armenian Hamster IgG-HRP, Santa Cruz Biotechnology, Cat: sc-2789, 1:500 dilution for IHC.
36. HRP-conjugated Goat anti-Rabbit IgG Secondary Antibody, Thermo Fisher Scientific, Cat: 31460, 1:3000 dilution for WB.
37. HRP-conjugated Goat anti-Mouse IgG Secondary Antibody, Thermo Fisher Scientific, Cat: 31430, 1:3000 dilution for WB and 1:500 dilution for IHC.

Validation

All antibodies used in this study were validated by the manufacturers or prior studies. The detailed information is provided as below.
1. Rabbit monoclonal anti-human p63, Cell Signaling Technology, https://www.cellsignal.cn/products/primary-antibodies/p63-a-d2k8x-xp-174-rabbit-mab/13109

2. Rat monoclonal anti-Ki67, Thermo Fisher Scientific, https://www.thermofisher.cn/cn/zh/antibody/product/Ki-67-Antibody-clone-SolA15-Monoclonal/14-5698-82
3. Mouse monoclonal anti-human KRT4, Santa Cruz Biotechnology, https://www.scbt.com/p/cytokeratin-4-antibody-6b10
4. Mouse monoclonal anti-human Jagged1, Santa Cruz Biotechnology, https://www.scbt.com/zh/p/jagged1-antibody-e-12
5. Armenian Hamster monoclonal anti-mouse Jagged2, BioLegend, https://www.biolegend.com/en-gb/products/purified-anti-mouse-jagged-2-antibody-5320
6. Mouse monoclonal anti-human ITGA6, Santa Cruz Biotechnology, https://www.scbt.com/p/integrin-alpha6-antibody-f-6?requestFrom=search
7. Mouse monoclonal anti-human ITGB1, Santa Cruz Biotechnology, https://www.scbt.com/p/integrin-beta1-antibody-4b7r?requestFrom=search
8. Mouse monoclonal anti-rat PKC zeta, Santa Cruz Biotechnology, https://www.scbt.com/p/pkc-zeta-antibody-h-1
9. Mouse monoclonal PE anti-human CD104 (ITGB4), BioLegend, https://www.biolegend.com/en-gb/products/pe-anti-human-cd104-antibody-4121
10. Rat monoclonal anti-E-cadherin, Sigma-Aldrich, https://www.sigmaaldrich.cn/CN/zh/product/sigma/u3254
11. Chicken polyclonal anti-KRT5, BioLegend, https://www.biolegend.com/en-gb/products/keratin-5-polyclonal-chicken-antibody-purified-10957
12. Rat monoclonal Purified anti-mouse F4/80, BioLegend, https://www.biolegend.com/en-gb/products/purified-anti-mouse-f4-80-antibody-4064
13. Mouse monoclonal Purified anti-human CD68, BioLegend, https://www.biolegend.com/en-gb/products/purified-anti-human-cd68-antibody-19975
14. Rat monoclonal Purified anti-mouse Ly-6G, BioLegend, https://www.biolegend.com/en-gb/products/purified-anti-mouse-ly-6g-antibody-4767
15. Rat monoclonal Purified anti-mouse CD4, BioLegend, https://www.biolegend.com/en-gb/products/purified-anti-mouse-cd4-antibody-252
16. Rat monoclonal Purified anti-mouse CD8a, BioLegend, https://www.biolegend.com/en-gb/products/purified-anti-mouse-cd8a-antibody-157
17. Rat monoclonal anti- ZO-1, Santa Cruz Biotechnology, https://www.scbt.com/p/zo-1-antibody-r40-76?requestFrom=search
18. Mouse monoclonal anti-SMA, Santa Cruz Biotechnology, https://www.scbt.com/p/smooth-muscle-actin-antibody-b4?requestFrom=search
19. Mouse monoclonal anti- human KRT14, Thermo Fisher Scientific, https://www.thermofisher.cn/cn/zh/antibody/product/Cytokeratin-14-Antibody-clone-LL002-Monoclonal/MA5-28125
20. Mouse monoclonal anti-Notch1, Santa Cruz Biotechnology, https://www.scbt.com/p/notch-1-antibody-a-8?requestFrom=search
21. Rat monoclonal anti-SOX2, Thermo Fisher Scientific, https://www.thermofisher.cn/cn/zh/antibody/product/SOX2-Antibody-clone-Btjce-Monoclonal/14-9811-82
22. Mouse monoclonal anti-GAPDH, Proteintech, https://www.ptgcn.com/products/GAPDH-Antibody-60004-1-Ig.htm
23. Rabbit monoclonal anti-Cleaved Notch1 (NICD1), Cell Signaling Technology, https://www.cellsignal.cn/products/primary-antibodies/cleaved-notch1-val1744-d3b8-rabbit-mab/4147
24. Alexa Fluor 488 AffiniPure Donkey Anti-Rat IgG, Jackson ImmunoResearch Laboratories, https://www.jacksonimmuno.com/catalog/products/712-545-153
25. Alexa Fluor 488 AffiniPure Donkey Anti-Rabbit IgG, Jackson ImmunoResearch Laboratories, https://www.jacksonimmuno.com/catalog/products/711-545-152
26. Alexa Fluor 488 AffiniPure Donkey Anti-Mouse IgG, Jackson ImmunoResearch Laboratories, https://www.jacksonimmuno.com/catalog/products/715-545-151
27. Alexa Fluor 488 AffiniPure Goat Anti-Armenian Hamster IgG, Jackson ImmunoResearchLaboratories, https://www.jacksonimmuno.com/catalog/products/127-545-099
28. Cy3 AffiniPure Donkey Anti-Mouse IgG, Jackson ImmunoResearch Laboratories, https://www.jacksonimmuno.com/catalog/products/715-165-151
29. Cy3 AffiniPure Donkey Anti-Rat IgG, Jackson ImmunoResearch Laboratories, https://www.jacksonimmuno.com/catalog/products/712-165-150
30. Cy3 AffiniPure Donkey Anti-Rabbit IgG, Jackson ImmunoResearch Laboratories, https://www.jacksonimmuno.com/catalog/products/711-165-152
31. Cy5 AffiniPure Donkey Anti-Mouse IgG, Jackson ImmunoResearch Laboratories, https://www.jacksonimmuno.com/catalog/products/715-175-151
32. Cy5 AffiniPure Donkey Anti-Rat IgG, Jackson ImmunoResearch Laboratories, https://www.jacksonimmuno.com/catalog/products/712-175-153
33. Cy5 AffiniPure Donkey Anti-Rabbit IgG, Jackson ImmunoResearch Laboratories, https://www.jacksonimmuno.com/catalog/products/711-175-152
34. Cy5 AffiniPure Donkey Anti-Chicken IgG, Jackson ImmunoResearch Laboratories, https://www.jacksonimmuno.com/catalog/products/703-175-155
35. Mouse anti-Armenian Hamster IgG-HRP, Santa Cruz Biotechnology, https://www.scbt.com/p/mouse-anti-armenian-hamster-igg-hrp
36. HRP-conjugated Goat anti-Rabbit IgG Secondary Antibody, Thermo Fisher Scientific, https://www.thermofisher.cn/cn/zh/antibody/product/Goat-anti-Rabbit-IgG-H-L-Secondary-Antibody-Polyclonal/31460
37. HRP-conjugated Goat anti-Mouse IgG Secondary Antibody, Thermo Fisher Scientific, https://www.thermofisher.cn/cn/zh/antibody/product/Goat-anti-Mouse-IgG-H-L-Secondary-Antibody-Polyclonal/31430

Animals and other research organisms

Policy information about studies involving animals; ARRIVE guidelines recommended for reporting animal research, and Sex and Gender in Research

Laboratory animals	Mice aged between 6 and 12 weeks were utilized in the studies. Both males and females were used. Mice were kept on the C57BL/6 and 129SvEv mixed backgrounds. Mouse strains included p63CreERT2, Jag1loxp/loxp, Jag2loxp/loxp, and wild type. All mice were allowed free access to food and water, were housed under 12-hour light and dark cycles at room temperature (20–24°C) with 40–60% humidity .
Wild animals	No wild animals were used.
Reporting on sex	Both male and female mice were used.
Field-collected samples	No field-collected samples were used.
Ethics oversight	All experimental procedures were conducted in accordance with protocols approved by the Institutional Animal Care and Use Committee (IACUC) of Shanghai Jiao Tong University.

Note that full information on the approval of the study protocol must also be provided in the manuscript.
